# Unsupervised synchronization of molecular dynamics trajectories via graph embedding and time warping

**DOI:** 10.1093/bioinformatics/btag017

**Published:** 2026-01-22

**Authors:** Manuel Mangoni, Salvatore Daniele Bianco, Francesco Petrizzelli, Michele Pieroni, Pietro Hiram Guzzi, Viviana Caputo, Tommaso Biagini, Tommaso Mazza

**Affiliations:** Department of Experimental Medicine, Sapienza University of Rome, Rome, 00185, Italy; UOS Computational Biology and Bioinformatics, Fondazione Policlinico Universitario A. Gemelli, IRCCS, Rome, 00168, Italy; UOS Computational Biology and Bioinformatics, Fondazione Policlinico Universitario A. Gemelli, IRCCS, Rome, 00168, Italy; Bioinformatics Laboratory, IRCCS Casa Sollievo della Sofferenza, S. Giovanni Rotondo, 71013, Italy; Department of Biochemical Sciences ‘A. Rossi Fanelli’, Sapienza University of Rome, Rome, 00185, Italy; Department of Surgical and Medical Sciences, University ‘Magna Græcia’ of Catanzaro, Catanzaro, 88100, Italy; Department of Experimental Medicine, Sapienza University of Rome, Rome, 00185, Italy; UOS Computational Biology and Bioinformatics, Fondazione Policlinico Universitario A. Gemelli, IRCCS, Rome, 00168, Italy; UOS Computational Biology and Bioinformatics, Fondazione Policlinico Universitario A. Gemelli, IRCCS, Rome, 00168, Italy

## Abstract

**Motivation:**

Molecular dynamics (MD) simulations provide detailed atomistic insights into biomolecular processes, but comparing independent trajectories remains challenging due to stochastic divergence. Misaligned simulations can obscure shared mechanisms or exaggerate differences, limiting reproducibility and mechanistic interpretation. A generalizable, unsupervised method for synchronizing and comparing MD trajectories across systems and conditions is, therefore, needed.

**Results:**

We introduce NetMD, a computational framework that synchronizes and analyzes MD trajectories by integrating graph-based representations with dynamic time warping. Trajectory frames are converted into residue-contact graphs, entropy-filtered to retain variable interactions, and embedded as low-dimensional vectors. NetMD aligns these vectorized trajectories through time-warping barycenter averaging, generating a consensus trajectory while pruning outlier simulations. Applied to transporters, demethylases, and large protein complexes relevant to neurological disease pathways and cancer, NetMD revealed shared multiphase dynamics and identified mutation- or ligand-specific deviations. This unsupervised, time-resolved approach enables direct comparison of MD ensembles across heterogeneous conditions. NetMD is robust and broadly applicable, providing a tool for uncovering conserved patterns and critical divergences in biomolecular dynamics.

**Availability and implementation:**

NetMD is freely available at https://github.com/mazzalab/NetMD.

## 1 Introduction

Molecular dynamics (MD) simulations have significantly advanced our understanding of atomic and molecular behavior (Karplus and McCammon 2002), enabling a comprehensive exploration of the structural properties and dynamic mechanisms of complex systems in biology, chemistry, and materials science. In biology, MD is a foundational computational genomics tool for elucidating the structural implications of genetic variants in molecular frameworks, thereby enhancing our understanding of genetic disorders and providing critical insights for research and diagnostic applications (Biagini *et al.* 2018, Ponzoni and Bahar 2018, Galano-*et al.* 2021). In chemistry, ab initio MD, such as the Car–Parrinello method, makes it possible to simulate complex bond-breaking and bond-forming reactions and effectively explore reactive potential energy surfaces effectively (Iannuzzi *et al.* 2003). MD has been widely applied in materials science to the study of Ti–Al-based alloys, providing insights into microstructural changes, alloy behavior, and interface phenomena (Li *et al.* 2024), as well as probing atomic-scale phenomena in materials, such as phase-change behavior for energy storage (Tafrishi *et al.* 2022) and guiding the design and characterization of novel nanomaterials (Islam *et al.* 2025).

Across these disciplines, the ability to compare multiple MD trajectories is central to identifying conserved mechanisms, quantifying variability, and distinguishing condition-specific behaviors. This is as true for detecting subtle differences in mutant protein folding as it is for evaluating alternative catalytic intermediates or benchmarking structural stability in engineered materials. However, comparing independent trajectories remains a challenge because stochastic divergence can obscure genuine mechanistic differences. Misaligned simulations may mask shared dynamic motifs or exaggerate differences, thereby limiting the reliability of the conclusions drawn from them. Robust trajectory comparison is therefore a critical step in deriving consistent, scientifically meaningful interpretations from MD data, regardless of whether the target system is a membrane transporter, catalytic complex, or nanostructured alloy.

Traditional methods for analyzing MD trajectories often rely on root-mean-square deviation (RMSD) calculations, quantitative comparisons of structural ensembles, and clustering techniques to compare different conformations. Although these approaches can effectively organize structures, their cluster definitions typically hinge on tunable RMSD thresholds and do not address asynchronous timing; they group “where” conformations are similar, not “when” different replicas reach comparable states. In this vein, recent pipelines such as *cc_analysis/Encodermap* (Hunkler *et al.* 2023) combined with HDBSCAN (McInnes *et al.* 2017) and MDSCAN (González-Alemán *et al.* 2022) remain fundamentally RMSD-driven despite algorithmic optimization.

Methods, such as those based on stochastic synchronization, can address the alignment of trajectories within similar systems or configurations. These methods depend heavily on shared noise sequences or initial conditions, features often absent in distinct molecular systems with divergent structures and dynamics. In fact, as shown by Uberuaga *et al.* (2004) and Sindhikara *et al.* (2009), identical random seeds in canonical dynamics can induce spurious lock-step motion; methods that compare trajectories frame by frame may mistake artificial correlations for genuine physical synchronization, unless they explicitly correct for timing differences using time warping. As such, they are ill-suited for identifying points in time where trajectories converge, or “meet,” to reveal shared patterns or critical events. Ensemble-level tools (e.g. ENCORE) quantify distribution overlaps from full RMSD matrices and thus provide global similarity rather than temporal alignment; they can also be computationally heavy and sensitive to non-equilibrated ensembles (Genheden and Ryde 2012, Tiberti *et al.* 2015).

A different class of methods focuses on system-specific kinetics and potentials of mean force (PMFs) in preselected collective-variable (CV) spaces, that is, Markov state models (MSMs) for a defined allosteric process, photodynamic pipelines with hand-picked internal coordinates, or string-method, swarms, and umbrella workflows. These are powerful within scope, but they target a different problem than unsupervised synchronization across heterogeneous MD runs (Meng *et al.* 2016, Fajer *et al.* 2017, Acheson and Kirrander 2023, Alisaac 2025).

In fact, temporal synchronization is not merely about aligning trajectories but requires uncovering consensus points of behavior that may arise from different initial states and pathways. Recent advancements in this field include classification of ligand-unbinding pathways. In (Ray and Parrinello 2024), the authors used the dynamic time-warping method to effectively align the MD trajectories. However, this technique is computationally demanding for high-dimensional datasets, exhibiting limited scalability to larger molecular systems or long simulation timescales. Moreover, the protocol incorporates *a priori* knowledge at the feature level; alignment and clustering operate on user-specified descriptor families (e.g. coordination numbers parameterized by a fixed switching function and length scale), and related implementations employ supervised CV construction to shape the latent space (Bonati *et al.* 2020).

The problem still lies with other types of dynamics, for example, the conformational changes between the open-closed states of membrane proteins, and is further compounded when dealing with mutant proteins, whose dynamics are unknown in advance because variations in sequence and structure may lead to significant differences in folding pathways, transition states, and interaction dynamics. Membrane proteins frequently undergo asynchronous multistep transitions that combine small helical shifts with large-loop rearrangements. Such complexity makes the global RMSD or simple contact-based comparisons particularly insensitive to the functional motions involved.

Here, we present NetMD, a generalizable framework for synchronizing and comparing MD trajectories. NetMD represents each simulation frame as a residue–contact graph, encodes its dynamic evolution through graph embeddings, and aligns multiple replicas using dynamic time warping (DTW) to obtain a consensus trajectory. By clustering aligned embeddings, the method identifies condition-specific dynamic signatures and detects time points of divergence across systems. This unsupervised approach enables consistent, time-resolved comparison of MD ensembles without prior labeling or system-specific tuning.

## 2 Materials and methods

**Figure 1:**
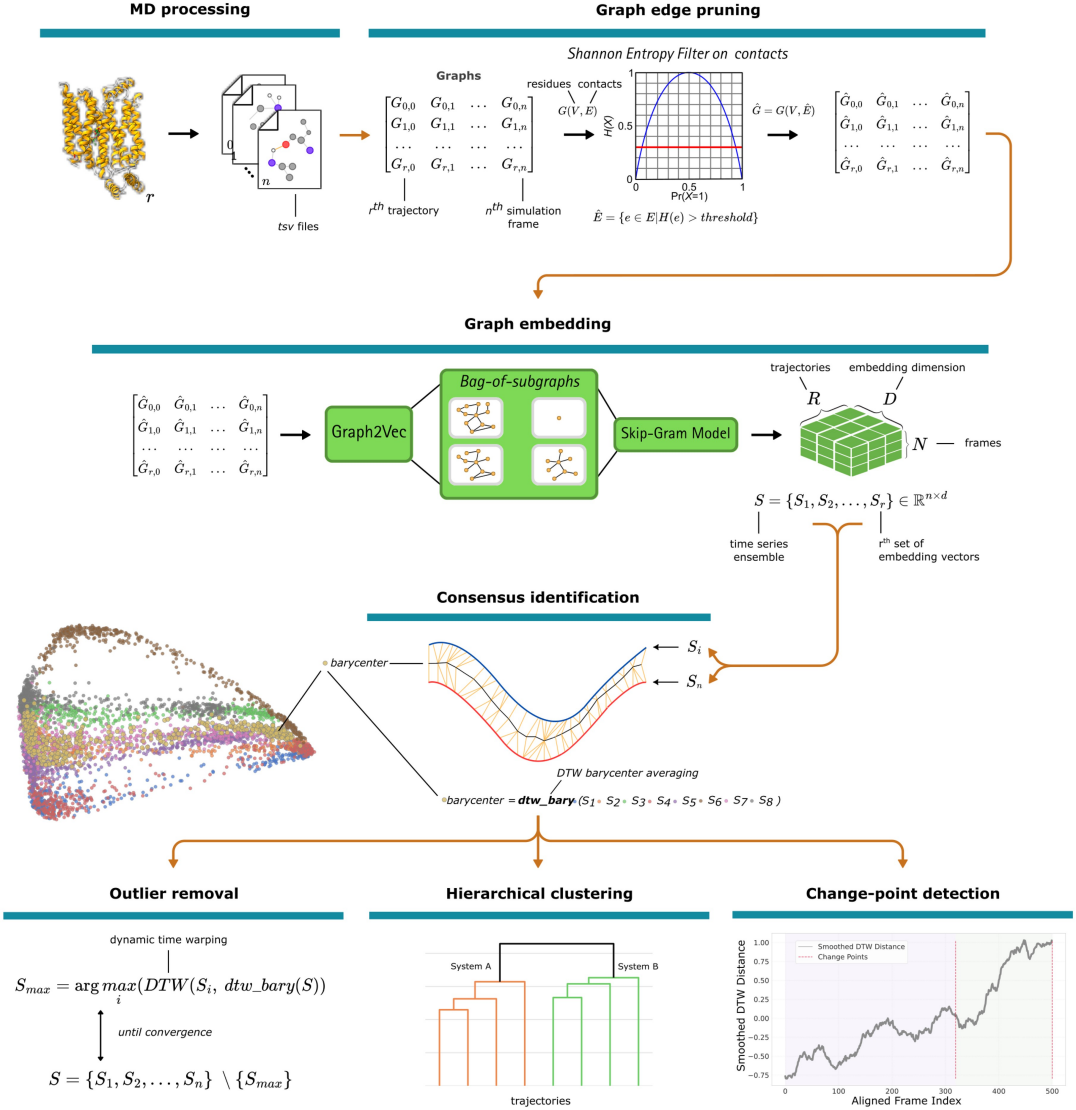
NetMD workflow. MD frames are converted into residue-contact graphs with entropy-filtered edges, embedded via a 16-dimensional Graph2Vec model into vectors, and aligned across replicas using DBA (normalized by series length). Divergent trajectories are pruned, and the clustered frames yield a state-transition network. Embeddings from the same system or under different conditions are compared using Ward’s hierarchical clustering to identify system-specific dynamic signatures. Change point detection is performed on the best replicas per system to highlight diverging states.

The overall workflow of NetMD is summarized in Fig. 1, which outlines the sequential steps from MD trajectory processing to graph-based embedding, alignment, and comparative analysis. The following sections describe the simulation datasets, graph-construction procedures, embedding and alignment algorithms, and subsequent analyses used to extract and interpret dynamic signatures across systems. Extended methods and additional figures are provided in the Supplementary Information.

### 2.1 Molecular dynamics simulations

Four representative systems were selected to cover diverse molecular contexts and simulation strategies, ranging from atomistic to coarse-grained (CG) representations.

- GLUT1 transporter dynamics were investigated using a hybrid Supervised Molecular Dynamics (SuMD) and Targeted Molecular Dynamics (TMD) protocol. SuMD was used to guide D-glucose into the binding cavity of the transporter, whereas TMD simulated the conformational transitions between outward-open, outward-occluded, and inward-open states. Systems included *wild-type* and mutant forms (R333Q and R333W), each embedded in a POPC lipid bilayer and neutralized in explicit solvent. Eight replicas of WT and R333Q and nine replicas of R333W were run under the Amber ff14SB force field.
- KDM6A simulations employed Gaussian-accelerated MD (GaMD) to probe the conformational dynamics of the catalytic Jumonji (JmjC) domain in complex with the H3 histone peptide. *Wild-type* and a Kabuki-syndrome-associated variant (R1255W) systems were each simulated in quintuplicate for 250 ns using Amber ff14SB and ZAFF parameters for the catalytic Zn(II) center.
- Mitochondrial Complex I was analyzed using CG MD trajectories representing *wild-type*, single-mutant, and triple-mutant assemblies. These data, available from (Rigobello *et al.* 2024), were modeled using reduced backbone-level contacts around the ND6 transmembrane helix to probe mutation-dependent conformational transitions.
- Finally, GLUT1–inhibitor systems were explored using Su-GaMD, an integrated scheme combining SuMD and GaMD to simulate full ligand-recognition pathways. Two inhibitors, cytochalasin B and phenylalanine amide, were each simulated in triplicate with 600 ps SuMD guidance followed by 100 ns GaMD sampling. All simulations were executed with Amber 22, following identical minimization, heating, and equilibration procedures.

### 2.2 Contact-graph construction and entropy filtering

Trajectory frames were converted into residue–contact graphs using GetContacts (https://getcontacts.github.io), which enumerates all residue–residue interactions within a cutoff distance for each frame. For CGsystems, nodes corresponded to residues within 15 Å of mutation sites, and edges were drawn between nodes closer than 6 Å.

To enhance signal-to-noise ratio, we applied an entropy-based pruning strategy that quantified the Shannon entropy of each contact across all frames and replicas. Low-entropy (i.e. low variance) and high-entropy (ie, high variance) edges were discarded, retaining only contacts that displayed meaningful variability. This adaptive filter highlights residues involved in functionally relevant conformational rearrangements while reducing bias from either rigid or noisy interactions. Unless otherwise stated, we used a default Shannon-entropy cutoff of 0.1. This low threshold was chosen based on the empirical contact-occupancy distributions of our test systems so that only contacts that were effectively invariant across frames and replicas were removed, while contacts with intermediate occupancies associated with conformational transitions were retained. We also verified that moderate variations around this value did not qualitatively affect the alignment results, indicating that NetMD is robust to this parameter choice.

### 2.3 Graph embedding

Filtered contact graphs were embedded into a 16-dimensional latent space using the Graph2Vec algorithm (Narayanan *et al.* 2017) implemented in the Karate Club Python library. Graph2Vec, based on the Weisfeiler–Lehman (WL) graph kernel, summarizes the local and intermediate connectivity patterns of molecular graphs via iterative neighborhood aggregation. Three WL iterations were used, capturing subgraph information up to three bonds away. This number of iterations was chosen as a compromise between expressiveness and computational cost, as it already captures local and intermediate neighborhoods without excessively enlarging the WL vocabulary. Retaining 90% of the variance provides a denoised low-dimensional representation that preserves the dominant dynamical trends; DTW alignment was always performed in the original 16-dimensional embedding space. Each frame was thereby represented as a fixed-size embedding vector, generating a time-series matrix of size N × R×D (frames × replicas × embedding dimensions). Dimensionality reduction by principal-component analysis (retaining 90% variance) followed by spectral embedding was used for visualization.

### 2.4 Trajectory alignment and consensus identification

Because MD replicas often evolve asynchronously, direct frame-to-frame comparison is unreliable. To recover a unified reference trajectory, we applied DTW to align the embedding time series across replicas. An average (barycentric) trajectory was computed using DTW Barycenter Averaging (DBA) (Petitjean *et al.* 2011), which minimizes the average temporal distance between all replicas while accounting for variable lengths. Normalized DTW scores were used to assess replica similarity. Outlier trajectories deviating most from the barycenter were iteratively pruned to ensure that the final consensus captured only reproducible dynamic behavior.

### 2.5 Clustering and detection of dynamic signatures

Pairwise DTW distances among replicas were subjected to Ward’s hierarchical clustering, enabling the discrimination of systems or experimental conditions according to their time-resolved dynamic signatures. Two complementary data-driven approaches, the largest-gap and elbow criteria, were used to estimate the optimal number of clusters without prior specification. Aligned embeddings and their clusters defined the core state-transition network of each system, revealing distinct conformational regimes sampled during the simulations.

### 2.6 Change-point detection

To pinpoint intervals of structural divergence, we computed frame-wise Euclidean distances between each aligned replica and its nearest consensus trajectory. The resulting temporal deviation profiles were smoothed using a moving-average filter to suppress thermal noise. Change points were then identified using the Pruned Exact Linear Time (PELT) algorithm with a linearly penalized cost function, detecting statistically significant shifts in the underlying trend of the deviation series. These segments delineate transition regions or mutant-specific disruptions in the system’s dynamic landscape.

## 3 Results

We applied NetMD to four representative systems to demonstrate its ability to synchronize and compare trajectories across diverse molecular contexts.

### 3.1 GLUT1 transporter: alignment of *wild-type* and mutant trajectories reveals reproducible transport cycles

For human glucose transporter 1 (GLUT1), variants of which are associated with Glucose Transporter Type 1 Deficiency Syndrome, a neurological disorder of energy metabolism, we assessed whether NetMD could synchronize *wild-type* and mutant trajectories, discriminate their dynamic behaviors, and determine the time windows of mutant divergence.

Replicate trajectories for the GLUT1 systems varied modestly in length, mostly extended to ∼500 frames, whereas a few concluded up to 20 frames earlier. Before embedding, we applied an ensemble-specific Shannon entropy filter to each replica of the systems, that is, the *wild-type*, R333Q, and R333W, pruning low-variability, non-informative contacts while preserving the most characteristic interactions of each ensemble.

**Figure 2:**
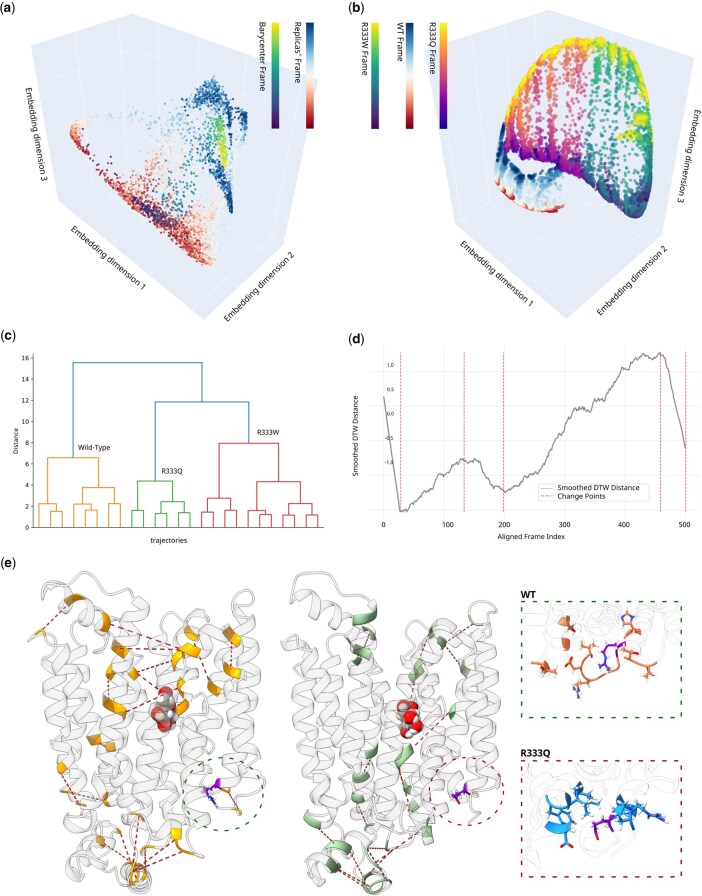
GLUT1 trajectory alignment and divergence via NetMD. (a) Three-dimensional embedding of *wild-type* GLUT1 transporter replicas overlaid with DTW barycenter trajectory. Each marker represents a single frame within the embedding space; replica frames are colored using a red–blue (RdBu) colormap with the color gradient encoding simulation time (from early to late frames), whereas the barycenter trajectory is shown using a viridis colormap, also progressing along time. (b) Combined 3D embeddings of the *wild-type* (WT) and Arg333 variants, R333Q and R333W, showing three distinct, non-overlapping clusters that reflect their divergent conformational dynamics. For each system, the color gradient encodes the temporal evolution of the corresponding trajectory: WT, R333Q, and R333W are colored with RdBu, plasma, and viridis colormaps, respectively. (c) Final hierarchical clustering dendrogram computed on the combined embeddings, illustrating the three major branches corresponding to the *wild-type*, R333Q, and R333W systems. (d) Change-point analysis of the aligned DTW distance time series for the two best replicas (one per system), with vertical dashed lines marking the detected transition shifts. (e) *Wild-type* versus mutant favored interactions observed during the change-point window, highlighted in 3D structures as dotted lines between the two interacting residues. On the left, top-favored *wild-type* contacts are shown in yellow; on the right, top-favored mutant interactions are shown in light green. The two panels on the far-right highlight contacts involving the mutant site: those more frequent in the *wild-type* (orange, top), and those more frequent in the mutant (blue, bottom). The mutant site was highlighted in violet. A list of the highlighted contacts is provided in Table 1, available as supplementary data at *Bioinformatics* online.

NetMD successfully synchronized and overlaid the time-series embeddings from both the *wild-type* and the two R333 mutant simulations. The resulting DTW barycenter traced a smooth trajectory through these states (Fig. 2a). In R333Q, only a single outlier required removal to achieve a uniform group (0.97 with respect to the 2nd worst of 0.78). In R333W, multiple pruning steps were necessary before the remaining replicas converged into a cohesive cluster (while the most representative replica aligned closely with the barycenter (score of 0.52), the four most deviant replicas had distances ranging from 0.78 to 0.94); the R333W variant disrupted the WT‑targeted simulation, leading to inconsistent sampling throughout the simulation.

To further delineate replica relationships, we performed hierarchical clustering on the DTW distance matrices. Coherently with embedding visualization (Fig. 2b), it distinguished *wild-type* from R333 variants (Fig. 2c), demonstrating that dynamic embeddings alone suffice to distinguish variant‑specific behaviors without prior labels.

The change‑point times identified by PELT fell precisely within the expected conformational‑rearrangement window and aligned with the phases highlighted by *k‑means* segmentation (Fig. 2d). When we examined the underlying contact patterns at these time points (Fig. 2e), focusing on interactions near the R333 site, we observed transient changes in contact frequencies that perfectly matched the deviations flagged in the PELT analysis, confirming that these were genuine structural events rather than noise.

### 3.2 KDM6A-H3 complex: GaMD alignment distinguishes *wild-type* and pathogenic variant dynamics

Lysine-specific demethylase 6A (KDM6A) is a globular protein that catalyzes the demethylation of tri/di-methylated histone H3, which is involved in Kabuki syndrome and various cancers, including bladder cancer, pancreatic cancer, and others (Petrizzelli *et al.* 2020, Biagini *et al.* 2022).

All KDM6A GaMD replicas comprised exactly 2500 frames each; given the initially dense contact graphs for KDM6A, with many edges present across most frames and replicas, we raised the Shannon entropy cutoff to 0.3 to selectively prune any ubiquitous and invariant interaction.

This adjustment reduced the ensemble bias by removing largely constant contacts while preserving the more informative, mutation-sensitive interactions that distinguished the *wild-type* and mutant simulations.

NetMD alignment of *wild-type* and a selected Kabuki syndrome-associated variant trajectories revealed a common core pathway in the *wild-type* trajectories and a broader embedding dispersion in the mutant (Fig. 1a and b, available as supplementary data at *Bioinformatics* online). The normalized DTW distances from the barycenter among *wild-type* replicas ranged from ∼0.82 for the closest runs up to 1.36 for the most divergent, underlining both highly representative trajectories and clear outliers, whereas in the mutant ensemble, distances spanned from roughly 0.84 up to 1.45, further underscoring the greater heterogeneity introduced by the variant.

We further quantified this by computing the full pairwise normalized DTW distance matrix, which showed that *wild-type* replicates clustered tightly (average inter-replica distance ∼2.16), whereas distances involving the R1255W variant spanned a much wider range (∼2.15 up to 4.06, mean ∼3.06), confirming increased heterogeneity in the mutant ensemble. Moreover, the average cross-group distance between *the wild-type* and mutant replicas was ∼3.5, which was substantially higher than either the *wild-type* or mutant self-distances, underscoring the clear separation between the two dynamic ensembles.

Embeddings from the mutant were grouped into clusters that were clearly separate from those of the *wild-type* simulations, faithfully reflecting the known perturbation of histone H3 binding and the resulting alterations in domain motion (Petrizzelli *et al.* 2020, Biagini *et al.* 2022) (Fig. 1c, available as supplementary data at *Bioinformatics* online). Change-point detection highlighted the intervals at which mutant and *wild-type* trajectories diverged (Fig. 1d, available as supplementary data at *Bioinformatics* online). We focused our contact analysis on change-point intervals where the distances exhibited pronounced increases. Within these windows, even without a predefined target event, we observed significant contact-frequency discrepancies at the mutation site and its immediate surroundings, highlighting the sensitivity of the methodology to subtle dynamic variations (Fig. 1e, available as supplementary data at *Bioinformatics* online). This application illustrates NetMD’s ability to align *wild-type* and variant trajectories and pinpoint divergence windows in longer, non-targeted simulations.

### 3.3 Mitochondrial Complex I: coarse-grained simulations capture mutation-dependent conformational heterogeneity

We further demonstrated the applicability of NetMD to large assemblies by analyzing CG MD trajectories of human mitochondrial Complex I, where each bead represents a group of atoms, and simplified backbone contact maps were sufficient to drive the synchronization and clustering process. These trajectories were not generated in the present work but were borrowed from (Rigobello *et al.* 2024), where the coarse-graining strategy and full simulation protocol are described in detail.

Each CG Complex I trajectory consisted of 5715 frames. Each Complex I ensemble (*wild-type*, single mutant, and triple mutant) was subjected to its own Shannon entropy filter, removing uniformly persistent backbone contacts and highlighting network changes driven by specific ND6 mutations around TMH3. Sparser contact graphs constructed using only backbone interactions within a 15 Å radius around the ND6 mutation sites captured the essential conformational transitions of TMH3 and showed distinct clustering of *wild-type*, single, and triple mutants. Embeddings reflected increasing divergence with mutation burden in both the embedding space and dendrogram (Fig. 2, available as supplementary data at *Bioinformatics* online), particularly for the triple mutant known to severely impair function, which was consistent with functional severity (Rigobello *et al.* 2024).

To quantitatively assess alignment quality, we computed the full pairwise normalized DTW distance matrix: in the *wild-type* ensemble, distances between replicates ranged from 1.09 to 1.38 (mean ≈ 1.20), indicating tight coherence; single-mutant distances spanned 1.07–1.22 (mean ≈ 1.15) and triple-mutant distances rose to 1.43–1.59 (mean ≈ 1.49), demonstrating substantially greater heterogeneity. Cross-group distances averaged ∼2.19 (*wild-type* versus single), ∼2.42 (*wild-type* versus triple), and ∼2.31 (single versus triple), with the triple-mutant replicates lying furthest from the *wild-type* ensemble, reflecting their more pronounced conformational divergence. The goal of this study was to capture and confirm the known distinctions between *wild-type* and mutant CG simulations at a reduced resolution, demonstrating the robustness of NetMD, even with simplified contact definitions.

### 3.4 GLUT1 inhibitor binding: Su-GaMD synchronization resolves ligand-specific conformational signatures

As a final proof of concept, we applied NetMD to comparative binding simulations of GLUT1 with two chemically distinct inhibitors, *cytochalasin B* and *phenylalanine amide*, where the trajectories comprised an initial variable-length SuMD phase followed by a fixed-length GaMD phase to test the capacity of the method for aligning asynchronous time series and resolving ligand-specific conformational signatures. This latter aspect is particularly relevant for guiding the rational design of more effective GLUT1 inhibitors, which are primary targets in many cancer treatments.

Before embedding, we applied the Shannon entropy filter separately to each inhibitor ensemble. This step pruned the contacts that showed minimal variability across all replicates for a given ligand, thereby emphasizing the most characteristic interactions of each binding pathway.

Each simulation concatenated a variable-length, 1000–5200 frames, SuMD simulation to a uniform-length, 2500 frames, GaMD simulation. This variability complicates the alignment and synchronization of time-series embeddings, which typically require comparable temporal scales to define a meaningful consensus trajectory. This was evident when mapping the DTW path on pairwise distance heatmaps, where the shortest trajectory was adaptively stretched, effectively “waiting” in embedding space until longer trajectories converged on the same conformational state. To mitigate this, we performed separate analyses of SuMD and GaMD segments.

**Figure 3:**
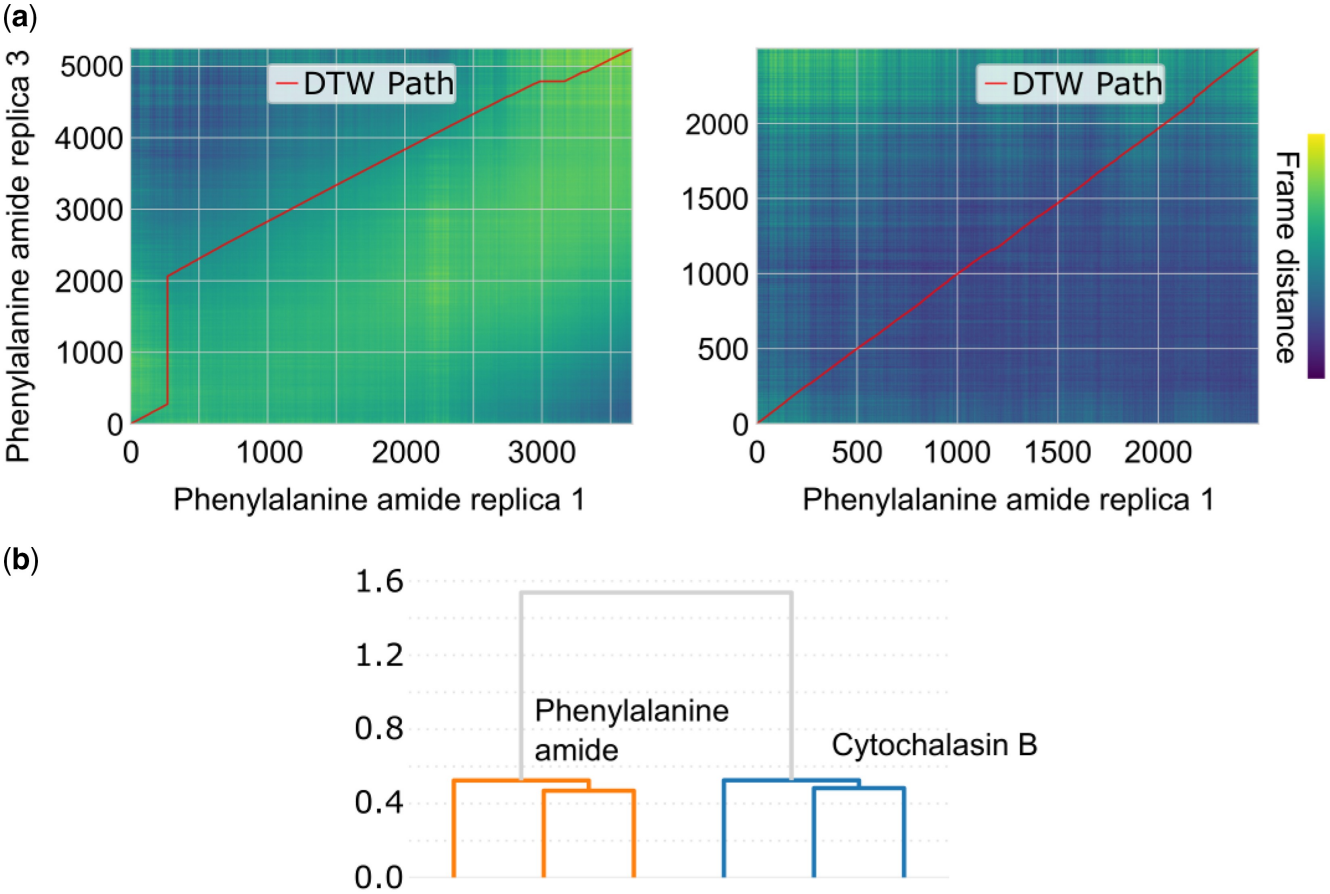
Dynamic time–warping alignments and clustering of GLUT1-phenylalanine amide simulation trajectories. (a) DTW alignments of ligand trajectories from SuMD (left) and GaMD (right) simulations. The heatmaps show the pairwise frame distances between two independent replicates (rep1 versus rep3) of the phenylalanine amide system, with the optimal DTW path overlaid in red. In SuMD, unequal trajectory lengths produce pronounced deviations of the red path from the diagonal (time warping), whereas in GaMD (in which all trajectories have the same length), the DTW path remains nearly linear. (b) Hierarchical clustering dendrogram: All SuMD+GaMD replicates from the phenylalanine amide system (orange) form one cluster, whereas those from cytochalasin B (blue) form the other.

Notably, the alignment reconverged toward the end of the SuMD phase, demonstrating that all replicates ultimately sampled the same key binding intermediates despite asynchronous sampling. In contrast, the GaMD segments were tightly aligned, with minimal adjustments (Fig. 3a). This consistency across replicates underscores that timing, rather than conformational heterogeneity, drives the initial alignment challenges. Alignment was quantified by computing the full pairwise normalized DTW distance matrix for all six replicates. Within‐inhibitor distances for cytochalasin B ranged from 1.87 to 2.50 (mean ≈ 2.13), and for phenylalanine amide from 1.71 to 2.37 (mean ≈ 2.14). Cross‐inhibitor distances averaged ∼2.36, which was substantially higher than either within‐group mean, highlighting the clear dynamic separation between the two binding pathways. Hierarchical clustering separated the inhibitors into distinct branches, thereby distinguishing the ligand-specific conformational pathways (Fig. 3b, Fig. 3, available as supplementary data at *Bioinformatics* online).

### 3.5 Benchmarking against descriptor-based DTW confirms NetMD sensitivity and clustering accuracy

We compared NetMD with the recent, reference synchronization method based on DTW by Ray and Parrinello (2024). Hence, we mirrored the Ray–Parrinello protocol by applying dependent-DTW directly to multivariate geometric descriptors. For each pair of GLUT1 trajectories (*A, B*), we computed a Cα-RMSD distance-matrix *M_ij_* (frame *i* in *A* versus frame *j* in *B*) and used *M* in dependent-DTW to obtain one inter-trajectory distance. Repeating this over all replicas yielded a DTW pairwise distance matrix. We then ran K-medoids clustering on that matrix (with silhouette analysis to select *K*). This comparison was performed for both GLUT1 *wild-type* and variant systems and ligands.

**Figure 4:**
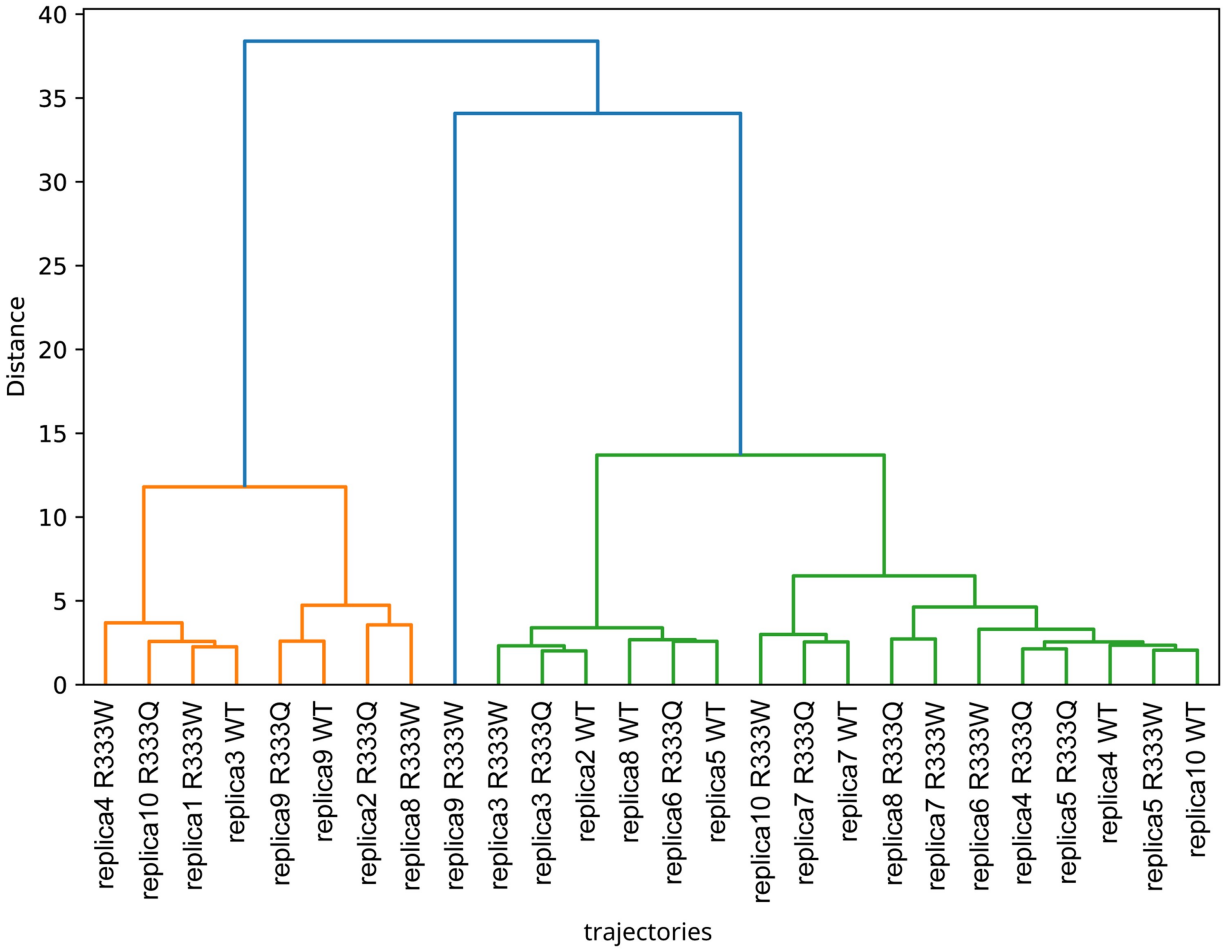
Hierarchical clustering using DTW distances of *wild-type*, R333Q, and R333W systems. It was computed with the descriptor-based method of (Ray and Parrinello 2024). Each leaf is a replica; the *y*-axis is the linkage distance between replicas.

NetMD distances were compact and self-organized into a clear block-diagonal heatmap with three tight clusters in the dendrogram, whereas the descriptor-only DTW baseline broadened distances, introduced off-diagonal hotspots/stripes most visibly among R333W replicas, and produced longer branches with earlier cross-group proximity, weakening WT–mutant separation (Figs 2c and 4, Fig. 4a, available as supplementary data at *Bioinformatics* online).

In the ligand-binding case (cytochalasin B versus phenylalanine amide), NetMD again yielded two clean 3 × 3 within-ligand blocks with a crisp bifurcation in the hierarchical tree, whereas the baseline was characterized by cross-group mixing that obscured ligand separation (Figs 3 and 4b, available as supplementary data at *Bioinformatics* online).

Across all tested systems, ranging from small transporters to large respiratory complexes and ligand–protein interactions, NetMD consistently aligned and clustered trajectories without prior labeling, resolved key conformational states, and mapped divergence windows to events of mechanistic significance.

## 4 Discussion

The results demonstrate that NetMD can uncover shared dynamic pathways and precisely localize divergence events across diverse molecular systems, linking algorithmic patterns directly to mechanistic insights. While our case studies focused largely on biomolecular contexts, the methodology is inherently generalizable. Any system whose evolution can be captured as a time series of interaction networks, from catalytic reaction intermediates in chemistry to defect propagation in crystalline solids, can, in principle, be analyzed using the same entropy-filtered graph embedding and time-warping framework. This flexibility arises from NetMD’s minimal reliance on system-specific prior knowledge, requiring only a consistent definition of contacts or interactions. NetMD therefore minimizes *a priori* specification compared to descriptor-led DTW pipelines that depend on user-defined descriptor families and, in related implementations, supervised CV construction.

The performance of NetMD across atomistic, coarse-grained, classical, and enhanced-sampling trajectories shows its capacity to handle datasets that vary widely in resolution, size, and duration. In biological applications, this enables the alignment of membrane transport cycles, globular enzyme conformational changes, and ligand-binding pathways. In chemistry, it could align reactive trajectories to identify conserved transition-state geometries or distinguish solvent-dependent mechanistic routes. In materials science, it could synchronize simulations of mechanical deformation, thermal annealing, or phase transitions to detect reproducible transformation sequences and isolate the effect of compositional variations.

By requiring only residue–contact graphs as input, the method remains agnostic to the simulation protocol, allowing its application to targeted simulations (e.g. SuMD), long non-targeted runs (e.g. GaMD), TMD, CG MD, or hybrid strategies without parameter reconfiguration. Meaningful alignments require that trajectories share a common node-set and broadly comparable overall folds, but they do not depend on identical initial contact graphs; static differences between systems primarily introduce an approximately constant offset in the embedding space rather than determining the warping path.

In each case study, NetMD not only synchronized trajectories but also produced interpretable divergence maps. For GLUT1 and KDM6A, the divergence windows coincided with key conformational transitions and contact rearrangements linked to functional impairment in the mutants. In the former case, in particular, NetMD revealed a three-stage dynamic segmentation across *wild-type* and mutants, which reconciled the glucose transport cycle, comprising an initial intake phase in which glucose approaches and binds from the extracellular side, a central conformational transition driving the outward-occluded to inward-open switch, and a final release phase where glucose exits toward the cytoplasm, directly from the aligned low-dimensional representations (Video 1, available as supplementary data at *Bioinformatics* online). In Complex I, increasing mutation burden correlated with greater dispersion in embedding space and larger cross-group DTW distances, consistent with experimental knowledge of functional severity. For GLUT1 inhibitors, NetMD resolved ligand-specific conformational signatures, even with asynchronous binding phases. For chemical and materials systems, analogous divergence points may correspond to bond rearrangements, nucleation events, or defect migration steps. The method’s unsupervised nature ensures that these events are detected without predefining reaction coordinates or state labels, supporting both hypothesis generation and validation.

Although all replicas conformed to the same global transition pattern, each showed subtle meaningful variations in the timing and intensity of individual dynamic phases, highlighting the sensitivity of the method in capturing fundamental shared mechanisms and divergent behaviors across simulations. In each case, the replicas formed tightly clustered trajectories following alignment; however, the mutants exhibited a greater deviation. This increased variation may reflect true alterations in the biophysical dynamics. Alternatively, this could result from the fact that mutant trajectories were supervised to realize the three conformational states common to the *wild-type* system. However, the mutants may partially or completely disrupt these states. In such cases, the simulation may struggle to reconcile the actual mutant-induced changes with the trajectory that it is “guided” to follow, resulting in irregular behaviors or alignment inconsistencies.

Although NetMD scales to long trajectories and large systems, computational demands grow with the number of frames and complexity of the WL graph vocabulary. The embedding time increases with the number of graphs and iterations, whereas the DTW alignment cost increases with sequence length and number of replicas. On the synthetic benchmarks reported in the online documentation, NetMD processed up to 8 replicas × 1500 frames of ∼450-node graphs in tens of minutes on a standard workstation, with peak memory usage on the order of 10–20 GBytes depending on graph density. For these problem sizes, runtime and memory scaled approximately linearly with the number of frames and replicas, and the dominant contribution to the cost was the Graph2Vec embedding step, with DTW and DBA adding only a small overhead. Parameter choices such as Shannon entropy cutoffs, number of WL iterations, and embedding dimensionality balance resolution against runtime. In the entropy-based contact pruning step, contacts that participate in transitions across replicas typically display intermediate occupancies and therefore high Shannon entropy and are retained, whereas only contacts that are almost always present or almost always absent across the ensemble are pruned. The entropy threshold is user-tunable, allowing users to adjust pruning stringency to the characteristics of their system and the type of transition they wish to analyze. Higher cutoffs and fewer iterations accelerate computation but may omit infrequent interactions; lower cutoffs and more iterations capture finer variations but increase the cost. The availability of a flexible API with multicore parallelization mitigates these trade-offs, enabling users to tailor performance to system complexity.

The versatility of NetMD suggests that it can serve as a unifying framework for comparative MD analysis across biology, chemistry, and materials science. By enabling an unsupervised, time-resolved comparison of complex molecular systems, it opens the door to systematic mining of dynamic signatures in systems as varied as enzymes, catalysts, nanomaterials, and functional polymers. As molecular simulations continue to expand in scope and resolution, such integrative approaches will be pivotal for translating dynamic patterns into mechanistic understanding, accelerating discovery, and informing predictive models across disciplines.

## Supplementary Material

btag017_Supplementary_Data

## Data Availability

All scripts and tutorials necessary to reproduce the analyses are freely available at https://github.com/mazzalab/NetMD, together with the processed residue–contact datasets used in this work. The documentation is available at https://mazzalab.github.io/NetMD. The residue–residue contact files extracted from the MD trajectories of the GLUT1, KDM6A, and GLUT1-inhibitors systems that were analyzed in this study are available at (Mangoni *et al.* 2025).

## References

[btag017-B1] Acheson K , Kirrander A. Automatic clustering of excited-state trajectories: application to photoexcited dynamics. J Chem Theory Comput 2023;19:6126–38. 37703098 10.1021/acs.jctc.3c00776 PMC10536988

[btag017-B2] Alisaac A. In silico analysis of quorum sensing modulators: insights into molecular docking and dynamics and potential therapeutic applications. PLoS One 2025;20:e0325830. 40489515 10.1371/journal.pone.0325830 PMC12148138

[btag017-B3] Biagini T , Chillemi G, Mazzoccoli G et al Molecular dynamics recipes for genome research. Brief Bioinform 2018;19:853–62. 28334084 10.1093/bib/bbx006

[btag017-B4] Biagini T , Petrizzelli F, Bianco SD et al KDM6A missense variants hamper H3 histone demethylation in lung squamous cell carcinoma. Comput Struct Biotechnol J 2022;20:3151–60. 35782738 10.1016/j.csbj.2022.06.041 PMC9232545

[btag017-B5] Bonati L , Rizzi V, Parrinello M. Data-driven collective variables for enhanced sampling. J Phys Chem Lett 2020;11:2998–3004. 32239945 10.1021/acs.jpclett.0c00535

[btag017-B6] Fajer M , Meng Y, Roux B. The activation of c-src tyrosine kinase: conformational transition pathway and free energy landscape. J Phys Chem B 2017;121:3352–63. 27715044 10.1021/acs.jpcb.6b08409 PMC5398919

[btag017-B7] Galano-Frutos JJ , García-Cebollada H, Sancho J. Molecular dynamics simulations for genetic interpretation in protein coding regions: where we are, where to go and when. Brief Bioinform 2021;22:3–19. 31813950 10.1093/bib/bbz146

[btag017-B8] Genheden S , Ryde U. Will molecular dynamics simulations of proteins ever reach equilibrium? Phys Chem Phys 2012;14:8662–77. 10.1039/c2cp23961b 22614001

[btag017-B9] González-Alemán R , Platero-Rochart D, Rodríguez-Serradet A et al MDSCAN: RMSD-based HDBSCAN clustering of long molecular dynamics. Bioinformatics 2022;38:5191–8. 36205607 10.1093/bioinformatics/btac666

[btag017-B10] Hunkler S , Diederichs K, Kukharenko O et al Fast conformational clustering of extensive molecular dynamics simulation data. J Chem Phys 2023;158:144109. 37061476 10.1063/5.0142797

[btag017-B11] Iannuzzi M , Laio A, Parrinello M. Efficient exploration of reactive potential energy surfaces using Car-Parrinello molecular dynamics. Phys Rev Lett 2003;90:238302. 12857293 10.1103/PhysRevLett.90.238302

[btag017-B12] Islam M , Rahman SMM, Mim JJ et al Applications of molecular dynamics in nanomaterial design and characterization—a review. Chem Eng J Adv 2025;22:100731.

[btag017-B13] Karplus M , McCammon JA. Molecular dynamics simulations of biomolecules. Nat Struct Biol 2002;9:646–52. 12198485 10.1038/nsb0902-646

[btag017-B14] Li N , Hao Z, Xu L et al A review of molecular dynamics simulation of different Ti-Al-Based alloys. Metals (Basel) 2024;14:1018.

[btag017-B15] Mangoni M , Biagini T, Petrizzelli F et al Synchronizing graph-embedded molecular dynamics trajectories via time-warping. 2025. 10.5281/ZENODO.15970688 (3 June 2025, date last accessed).

[btag017-B16] McInnes L , Healy J, Astels S. hdbscan: hierarchical density based clustering. JOSS 2017;2:205.

[btag017-B17] Meng Y , Shukla D, Pande VS et al Transition path theory analysis of c-src kinase activation. Proc Natl Acad Sci U S A 2016;113:9193–8. 27482115 10.1073/pnas.1602790113 PMC4995974

[btag017-B18] Narayanan A , Chandramohan M, Venkatesan R et al graph2vec: learning distributed representations of graphs. 2017. 10.48550/ARXIV.1707.05005, preprint: not peer reviewed.

[btag017-B19] Petitjean F , Ketterlin A, Gançarski P. A global averaging method for dynamic time warping, with applications to clustering. Pattern Recognit 2011;44:678–93.

[btag017-B20] Petrizzelli F , Biagini T, Barbieri A et al Mechanisms of pathogenesis of missense mutations on the KDM6A-H3 interaction in type 2 Kabuki syndrome. Comput Struct Biotechnol J 2020;18:2033–42. 32802275 10.1016/j.csbj.2020.07.013 PMC7412721

[btag017-B21] Ponzoni L , Bahar I. Structural dynamics is a determinant of the functional significance of missense variants. Proc Natl Acad Sci USA 2018;115:4164–9. 29610305 10.1073/pnas.1715896115 PMC5910821

[btag017-B22] Ray D , Parrinello M. Data-driven classification of ligand unbinding pathways. Proc Natl Acad Sci USA 2024;121:e2313542121. 38412121 10.1073/pnas.2313542121 PMC10927508

[btag017-B23] Rigobello L , Lugli F, Caporali L et al A computational study to assess the pathogenicity of single or combinations of missense variants on respiratory complex I. Int J Biol Macromol 2024;273:133086. 38871105 10.1016/j.ijbiomac.2024.133086

[btag017-B24] Sindhikara DJ , Kim S, Voter AF et al Bad seeds sprout perilous dynamics: stochastic thermostat induced trajectory synchronization in biomolecules. J Chem Theory Comput 2009;5:1624–31. 26609854 10.1021/ct800573m

[btag017-B25] Tafrishi H , Sadeghzadeh S, Ahmadi R. Molecular dynamics simulations of phase change materials for thermal energy storage: a review. RSC Adv 2022;12:14776–807. 35702228 10.1039/d2ra02183h PMC9112287

[btag017-B26] Tiberti M , Papaleo E, Bengtsen T et al ENCORE: software for quantitative ensemble comparison. PLoS Comput Biol 2015;11:e1004415. 26505632 10.1371/journal.pcbi.1004415 PMC4624683

[btag017-B27] Uberuaga BP , Anghel M, Voter AF. Synchronization of trajectories in canonical molecular-dynamics simulations: observation, explanation, and exploitation. J Chem Phys 2004;120:6363–74. 15267525 10.1063/1.1667473

