# Supplementary material for: Unsupervised synchronization of molecular dynamics trajectories via graph embedding and time warping
Source: Bioinformatics. 2026 Jan 22;42(2):btag017. doi: 10.1093/bioinformatics/btag017 (PMC12930375; doi:10.1093/bioinformatics/btag017)
Supplement: btag017_Supplementary_Data [file btag017_supplementary_data.zip › NetMD_SI_revised.docx]

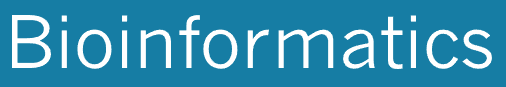


**Supporting Information for**

Unsupervised Synchronization of Molecular Dynamics Trajectories via Graph Embedding and Time Warping

Manuel Mangoni^1^, Salvatore Daniele Bianco^2^, Francesco Petrizzelli^3^, Michele Pieroni^4^, Pietro Hiram Guzzi^5^, Viviana Caputo^1^, Tommaso Biagini^2,*^, Tommaso Mazza^2^

Tommaso Biagini

Email: [tommaso.biagini@policlinicogemelli.it](mailto:tommaso.biagini@policlinicogemelli.it)

**This PDF file includes:**

Extended methods

Supplementary Figures S1-4

Legend for Video S1

Software availability

SI References

**Extended Methods**

**Molecular Dynamics Simulation**

We exploited four different simulation strategies, which span classical and enhanced sampling MD methods, to study as many real molecular systems.

**GLUT1 conformational transition during glucose translocation**

Supervised Molecular Dynamics (SuMD) [(1)](https://paperpile.com/c/dMCur1/xTZG) is an adaptive sampling technique that enables the rapid exploration of ligand-receptor recognition pathways on a significantly reduced timescale compared to standard MD approaches. Targeted molecular dynamics (TMD) [(2)](https://paperpile.com/c/dMCur1/CHSw6) approaches are frequently used in the MD field to induce conformational changes in a target structure using steering forces, overcoming energy barriers, and allowing more efficient conformational space exploration. Here, we extensively simulated the entire glucose pathway through its main transporter, GLUT1, in *wild-type* and mutant contexts, using a hybrid MD strategy. First, we implemented SuMD to quickly guide glucose into its central binding pocket, followed by TMD to simulate the conformational switching in the presence of glucose.

The initial outward-open conformation, together with the outward-occluded conformation, was modelled by homology modeling using the X-ray outward-open and outward-occluded conformations of GLUT3 as a template (respectively, PDB id: 4ZWB and 4ZW9), and then embedded into a lipid bilayer composed of 1-palmitoyl-2-oleoyl-glycero-3-phosphocholine (POPC) using CHARMM-GUI [(3)](https://paperpile.com/c/dMCur1/IpzRr) to simulate an accurate cellular environment *in-silico*. Conversely, the crystal structure of the GLUT1 inward-open configuration (PDB ID: 4PYP) was used as the final target for TMD simulations. Single point mutations Arg333Trp (R333W) and Arg333Gln (R333Q) were introduced using ChimeraX [(4)](https://paperpile.com/c/dMCur1/xS4rK). Both *wild-type* and mutant systems were inserted into a simulation box filled with TIP3P and Na+/Cl− counter ions to neutralize the overall charge. Finally, a D-glucose molecule was inserted into the simulation box far from the GLUT1 central cavity and slowly guided into the X-ray-determined binding pocket, while exploring a variety of potential binding pathways.

The obtained systems were energy-minimized by applying the steepest descent method, followed by the conjugate gradient method, and then gradually heated and equilibrated for 10 ns using a time step of 1 fs. Ten replicates of each simulation were conducted using the Amber ff14SB force field. The SuMD phase consisted of short consecutive simulations of 300 ps while the TMD phase was divided into two different run of 10 ns, the first with geometrical constraint that guided the outward-open→outward-occluded transition, while in second run we applied steering forces to drive both the outward-occluded→inward-open transition and the glucose along the Z-axis towards the cytoplasm. All simulations were performed using Amber 22 [(5)](https://paperpile.com/c/dMCur1/h22VX).

**Gaussian accelerated MD of KDM6A interaction with H3 histone**

Gaussian-accelerated Molecular Dynamics (GaMD) [(6)](https://paperpile.com/c/dMCur1/shQ3F) represents one of the most robust MD approaches to simulate protein conformational transitions among many biological processes. By adding a harmonic boost potential that follows a Gaussian distribution, GaMD expands sampling by orders of magnitude without the need for predefined reaction coordinates.

Here, we simulated the impact of a known pathogenic missense mutation, Arg1255Trp (R1255W), on KDM6A-H3 interaction. Specifically, the catalytic Jumonji (JmjC) domain of KDM6A, an histone demethylase, was simulated in complex with the H3 histone using GaMD, following the system preparation and simulation protocol illustrated in [(7)](https://paperpile.com/c/dMCur1/9MZCW). In brief, after standard minimization and equilibration steps, as described above, the *wild-type* and mutant systems were simulated five times for 250 ns each. The Amber ff14SB force field was used to parameterize the amino acids, whereas the Zinc AMBER force field (ZAFF) was employed for the Zn(II) ion. All simulations were performed using Amber 22 [(5)](https://paperpile.com/c/dMCur1/h22VX).

**Coarse-grained simulations of single and triple mutant mitochondrial CI complex**

Coarse-grained (CG) molecular dynamics simulations [(8)](https://paperpile.com/c/dMCur1/CXuUV) are widely used to simulate biomolecular systems on large time and size scales. In a CG simulation, groups of atoms are represented as a single particle, reducing the computational complexity while retaining the essential structural and dynamic properties of a biomolecule.

Here, we tested our NetMD approach on the CG-MD simulations described in [(9)](https://paperpile.com/c/dMCur1/1O1xL) and made available at [(10)](https://paperpile.com/c/dMCur1/s62HE). In this study, we employed a CG representation of mammalian respiratory complex I (CI) to determine the impact of putatively pathogenic mtDNA variants, alone or in combination, on the conformational transition of the transmembrane helix (TMH3) of MT-ND6, which carries the m.14484T>C primary mutation and is fundamental for CI function. We selected only human-open systems for the *wild-type*, single mutant, and triple mutant.

**Comparison of the binding mechanism of GLUT1 inhibitors employing Su-GaMD**

Rational integration of different MD sampling techniques is fundamental for the accurate characterization of complex biological events, such as ligand-induced activation/inhibition processes. Su-GaMD [(11)](https://paperpile.com/c/dMCur1/B4yu8) is an enhanced sampling technique that incorporates the SuMD and GaMD approaches described in previous sections, providing a powerful method to simulate the entire ligand recognition process together with the occurring receptor conformational changes, enabling a deeper understanding of the ligand mechanism of action.

Here, we compared the full binding mechanism of two known GLUT1 transport inhibitors, cytochalasin B and phenylalanine amide, whose complex structures with the transporter are available in the PDB (5EQI and 5EQG, respectively), using the Su-GaMD protocol described in [(11)](https://paperpile.com/c/dMCur1/B4yu8). GLUT1 was inserted into a POPC bilayer, and each inhibitor was initially placed approximately 50 Å away from the respective binding pocket. GaMD acceleration parameters were derived from prior GaMD simulations. Short 600 ps trajectories were employed for the SuMD steps, followed by 100 ns of GaMD. Thus, following the standard minimization and equilibration protocol described in Section 1.1, each system was simulated in triplicate using the Amber 14SB force field. All simulations were performed using Amber 22 [(5)](https://paperpile.com/c/dMCur1/h22VX).

### **MD trajectories processing**

**MD trajectory to Graph conversion**

From the raw molecular dynamics (MD) simulation trajectories, we derived time-resolved contact maps by generating edge lists that captured all residue–residue interactions across frames. Trajectories were processed using GetContacts [<https://getcontacts.github.io/>], which enabled the systematic extraction of pairwise residue interactions on a per-frame basis. For each frame, we recorded a list of interacting residue pairs, annotated with the corresponding frame index. This approach yielded a temporally indexed contact graph for each replica, an efficient yet expressive representation that preserved the full connectivity of the system while maintaining both spatial and temporal resolution. These contact graphs served as the foundation for the subsequent frame-by-frame analysis of interaction dynamics across replicas.

For the coarse-grained system, we first identified all residues that fell within a 15 Å radius of the mutation site in at least one frame. This spatially filtered node set was then used to build per‐frame edge lists for each replica. At every time point, an undirected edge was placed between any two of the selected residues whenever their pairwise distance dropped below 6 Å.

**Graph edge pruning**

The residue–contact graph was pruned using an entropy-based metric to enhance the representativeness of the dataset. For all frames of all simulation replicas of a molecular system, we calculated the Shannon entropy, a widely recognized metric for quantifying variability and disorder in biological systems. In this application, entropy provides an *ad hoc* method for capturing the dynamism of residue interactions, which is essential for understanding conformational flexibility and functional hotspots [(12)](https://paperpile.com/c/dMCur1/8M0h2). It then provides a quantitative measure of how interactions fluctuate over time.

This filter excludes (i) low-frequency noisy contacts and (ii) high-frequency invariant contacts that, while structurally stabilizing, provide little discriminative value between frames. The choice of a frequency threshold is critical because it aims to emphasize case-specific interaction patterns. Lower thresholds may capture more interactions, potentially increasing sensitivity to subtle variations. Higher thresholds provide stricter criteria and focus on only the most dynamic interactions. Nevertheless, the possibility of tuning this value ensures that the method can be tailored to specific biological questions or data characteristics.

After applying the entropy filter, we extracted subgraphs that contained only the retained edges and their incident nodes. In such graphs, nodes represent residues and edges denote the informative contacts between residues. Nodes were annotated using multiple features. We specifically used the position of the residue within the amino acid sequence as one such feature, which inherently captures information regarding both the primary and tertiary structures of the protein. This ensures that no information about the residue identity is lost during graph construction, thus maintaining a direct link to the biological entities of the molecule. Importantly, the number of nodes remained consistent across all graphs because they represent the same set of residues in the molecular system. This ensures that variations in graph properties are strictly related to changes in residue interactions over time and allows us to accurately capture the system’s behavior over time and across different replicas, providing residue–level insights [(13)](https://paperpile.com/c/dMCur1/aovXV).

### **Graph Embedding**

Embeddings were generated from the graphs using the Graph2Vec [(14)](https://paperpile.com/c/dMCur1/J8Ivy) method implemented in the Karate Club [(15)](https://paperpile.com/c/dMCur1/95qCT) Python library. Graph2Vec is a graph-embedding method inspired by the document-embedding approach Doc2Vec [(16)](https://paperpile.com/c/dMCur1/Cn6gi). It represents entire graphs as fixed-size vectors by learning a continuous vector space where structurally similar graphs are mapped closer together. Graph2Vec employs the Weisfeiler–Lehman (WL) kernel to summarize the graph topological patterns. At different scales, each node’s representation is updated by combining it with that of its immediate neighbors. Thus, after several rounds, the final representation progressively reflects larger neighborhoods (i.e., both direct and indirect contacts). This makes the WL kernel particularly well suited for molecular graphs, where the complex connectivity of residues must be precisely represented, and by the end of the process, the refined node representation encodes both local and intermediate structural features of the graph. After applying the WL kernel, the Graph2Vec algorithm partitions the graph into a collection of rooted subgraphs (*bag-of-subgraphs*), each of which corresponds to a node and its local neighborhood defined by the previous step. These subgraphs are the key elements for feature extraction, and a feature vector is constructed for each graph by counting the occurrences of different subgraph patterns and generating a distribution of subgraph labels.

Then, a skip-gram model is trained similar to the approach used in Word2Vec [(17)](https://paperpile.com/c/dMCur1/kpf6x), which learns to represent graphs as real-valued vectors by analyzing their surrounding context (*bag-of-subgraphs)*, thereby capturing the relationships between them, like how a language model learns word embeddings based on their context within sentences. Using this approach, Graph2vec can learn low-dimensional embeddings that encode the relationships and co-occurrence patterns of subgraph labels that reflect the topological properties of the graph.

In our workflow, we selected the following parameters for the Graph2Vec method: (i) the dimensionality of the embeddings was set to 16, balancing the need for a compact representation with the ability to capture meaningful structural patterns; and (ii) the number of WL iterations (*wl_iterations*) was set to three. This allows the model to incorporate subgraph information up to three hops away from each node, thereby capturing both local and intermediate graph structures. Additionally, the downsampling rate was fixed at zero, ensuring that no subgraph patterns were discarded during the embedding process. All other parameters were kept at their default values as they adequately met the needs of our analyses without requiring additional adjustments.

The embedding result was a three-dimensional matrix of size *NxRxD*, where *N* corresponds to the number of frames, 𝑅 to the number of replicas, and *D* to the embedding dimensions: more specifically, each graph, representing a single frame from a specific replica, was encoded as a vector of size *D*.

To facilitate the visualization of the embedding matrix, Principal Component Analysis (PCA) was used to retain 90% of the variance, reducing the dimensionality while preserving the most significant features of the data. This step ensures that the most important patterns in the embeddings are maintained, thereby allowing for a meaningful visual representation. Subsequently, Spectral Embedding was used to project the data into a low-dimensional space based on the connectivity structure of the graph. This two-step approach, PCA followed by Spectral Embedding, enhances the visualization of relationships and clustering among frames and replicas within the embedding space. Moreover, the analysis provided hints on how graphs group according to their structural similarities, providing a more intuitive interpretation of the data, and helping in the identification of distinct patterns or clusters.

### **Consensus Identification via Dynamic Time Warping**

We considered the set of embedding vectors from each replica as a time series of size *NxD*. Each embedding vector of dimension *D* encodes a snapshot of the conformation of the protein at a given frame in *N*, faithfully describing its dynamic behavior throughout the simulation. Consequently, to capture a consensus representation of the embeddings across all replicas, we employed the Dynamic Time Warping Barycenter Averaging (DBA) technique [(18, 19)](https://paperpile.com/c/dMCur1/jnRQQ+VCDZM).

DBA is a method for computing the barycenter of multiple time series while accounting for their temporal alignment, thereby providing a central representation of the data. It aims to identify a central trajectory that minimizes the average distance between time series while also considering their nonlinear temporal shifts, making it particularly suitable for data with varying lengths and misalignments. It is based on Dynamic Time Warping (DTW), which allows the comparison of time series with different lengths or temporal shifts. DTW identifies an optimal alignment by stretching or compressing segments of the time series to minimize the overall discrepancy between them. This alignment is crucial for capturing the true temporal dynamics of the system, even in the presence of distortions in the timing of individual time series.

Through this approach, we obtained a “*consensus embedding,*” which captures a generalized, stable pattern of the protein's dynamics across all replicas. However, in addition to computing the barycenter, we aligned all replicas to the consensus embedding. This step allowed us to normalize the dynamic behavior of each replica relative to the central representation, ensuring that all replicas were compared in a consistent temporal frame.

To measure the dissimilarities between each time series, we introduced a scoring function based on the normalized DTW distance. This function accounts for differences in the lengths and temporal shifts of the time series. The normalized DTW formula is as follows:


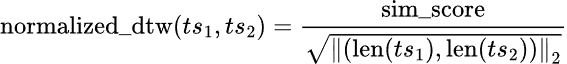


where *sim_score* is the similarity score obtained from the DTW path computation between time series *ts1* and *ts2* and the denominator normalizes the score by considering the lengths of the time series.

### **Data-Driven Cluster Detection and Outlier Removal**

To explore the relationships between replicas, we performed hierarchical clustering based on the pairwise distances computed between each time series. A linkage matrix was computed using Ward’s method to minimize the total within-cluster variance.

To identify the optimal number of time-series clusters, that is, the number of distinct groups exhibiting similar temporal patterns, we devised two complementary, data-driven strategies that do not require prior specification of the number of clusters. (i) The *largest gap method* detects the point where a substantial increase in linkage distances occurs, assuming that it represents a natural division between clusters. The reasoning behind this is that stepwise dendrograms frequently create higher jumps in linkage distances whenever its elements lack clear cluster affinity, which can serve as indicators for identifying the optimal number of clusters [(20)](https://paperpile.com/c/dMCur1/Kpvpw). (ii) The classical *elbow method* is based on the principle of diminishing returns in the cluster cohesion. It assesses the linkage distances between merges in the hierarchical clustering process and identifies the point at which adding more clusters no longer significantly reduces within-cluster variance. This point, known as the "elbow," marks the optimal balance between excessively fragmented data into too many clusters (oversegmentation) and grouping dissimilar elements into too few clusters (undersegmentation). By detecting the steepest decline in distances between successive merges, the method estimates the dendrogram's inflection point, enabling data-driven determination of optimal cluster partitioning.

Finally, we implemented an iterative pruning method to further refine the reference embedding. The main goal of this approach is to identify and eliminate replicas that deviate most significantly from the consensus, thereby ensuring that the final central representation robustly reflects the core dynamics. At each step, the barycenter was computed and the replica with the largest deviation was removed until only the two closest replicas (both at the same distance from the barycenter) remained. This process allowed us to progressively eliminate outliers and minimize the influence of extreme or atypical conformational states.

### **Change-Point Detection via Temporal Deviation Profiling**

To detect anomalous dynamic behaviors within individual replicas, we computed the pairwise Euclidean distance at each aligned time step (frame), comparing each replica to a reference trajectory selected as the closest to the consensus. This procedure generates a temporal deviation profile that captures the localized structural divergence over time.

To reduce noise and enhance the interpretability of these temporal distance signals, we applied a centered moving average filter with an odd-sized sliding window. MD data can exhibit high-frequency noise owing to intrinsic thermal fluctuations, sensitivity to initial velocities, finite sampling, and numerical integration artifacts. With this data, a moving average can smooth out these short-term irregularities.

We then used the Pruned Exact Linear Time (PELT) algorithm, an efficient change-point detection algorithm designed to identify points in a time series in which the underlying statistical properties change. PELT aims to determine a set of change points [
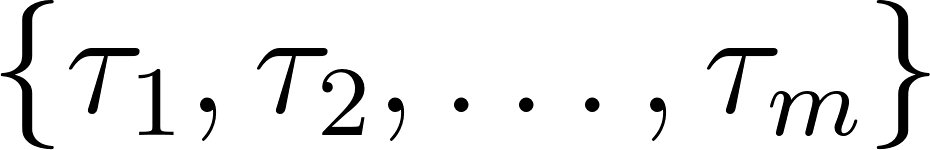
](https://www.codecogs.com/eqnedit.php?latex=%5C%7B%5Ctau_1%2C%20%5Ctau_2%2C%20%5Cldots%2C%20%5Ctau_m%5C%7D#0)that minimize the following objective function:


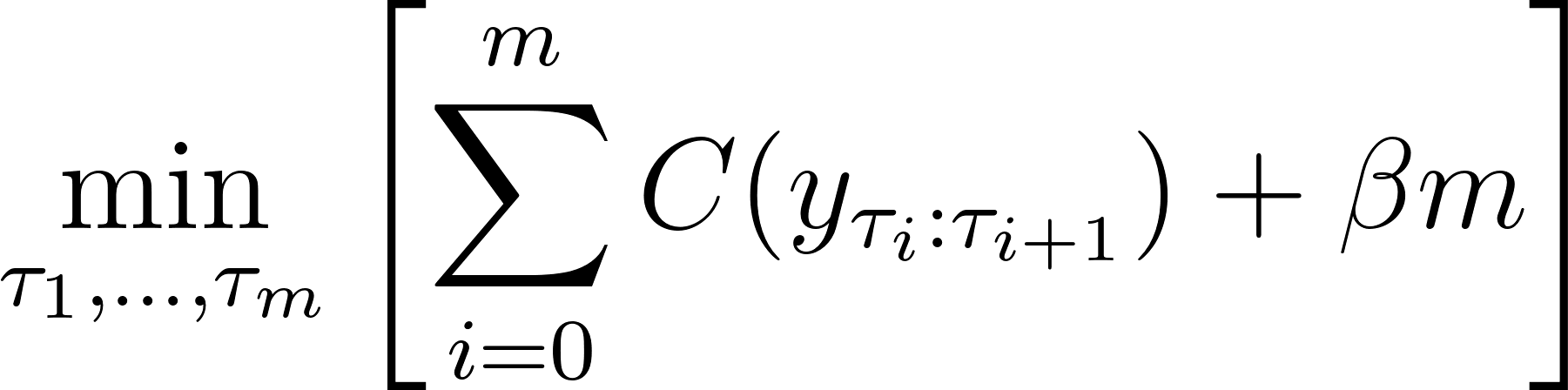


where i) *C* is the cost of each segment; ii) *β* is the penalty term; and iii) *m* is the number of change points. More specifically, we opted for a linear cost function that assumes that, within each segment, the observed signal can be described by a straight line (constant slope and intercept), while also penalizing deviations from that line, thereby allowing the algorithm to identify points at which the topology of the system changes significantly. Here, a “segment” is simply the interval between two successive change points, over which the deviation profile is treated as stationary and can be changed to tune the sensitivity accordingly: smaller values yield shorter and thus more segments by checking for mean shifts every few frames, whereas larger values produce fewer, longer segments. The penalty was logarithmically scaled with the number of frames, allowing for a consistent performance across datasets of varying sizes.

**Supplementary Figures**

**
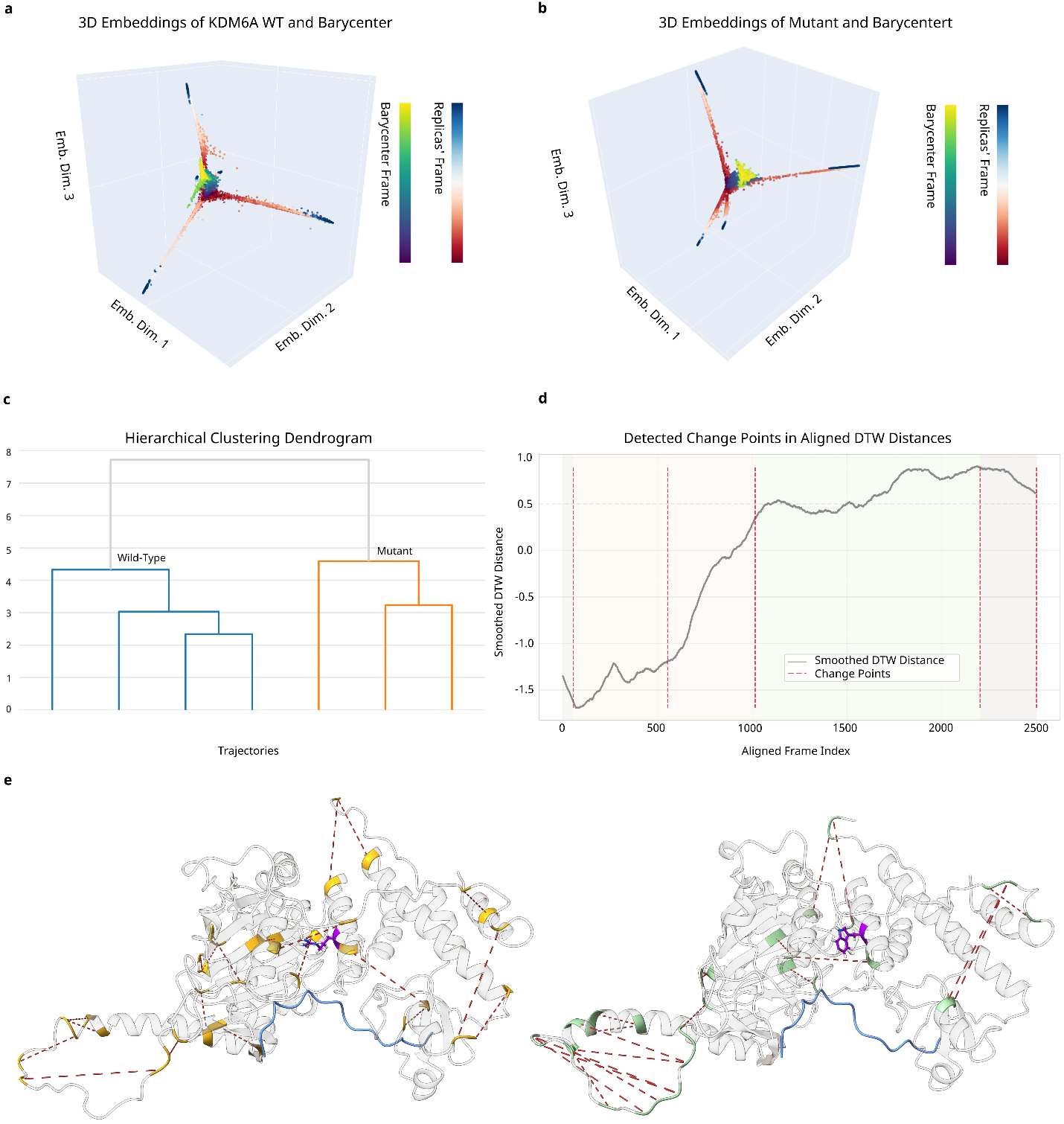
**

**Figure S1.** (a) Three-dimensional embedding of *wild-type* KDM6A, with the DTW barycenter trajectory overlaid (viridis). Each marker represents a single frame within the embedding space. Differences in embedding space represent the unsupervised nature of the simulations; (b) Three-dimensional embedding of the mutant KDM6A, with the DTW barycenter trajectory overlaid (viridis). (c) Final hierarchical clustering dendrogram computed on the combined embeddings, illustrating the two major branches corresponding to *wild-type* and the mutant. (d) Change-point analysis of the aligned DTW distance time series, with vertical dashed lines marking transition shifts. (e) *Wild-type* versus mutant favored interactions observed during the change-point window, highlighted in 3D structures as dotted lines between the two interacting residues. On the left, top-favored *wild-type* contacts are shown in yellow; on the right, top-favored mutant interactions are shown in light-green. The mutant site was highlighted in violet. A list of the highlighted contacts is provided in **Supplementary Table 2**.

**
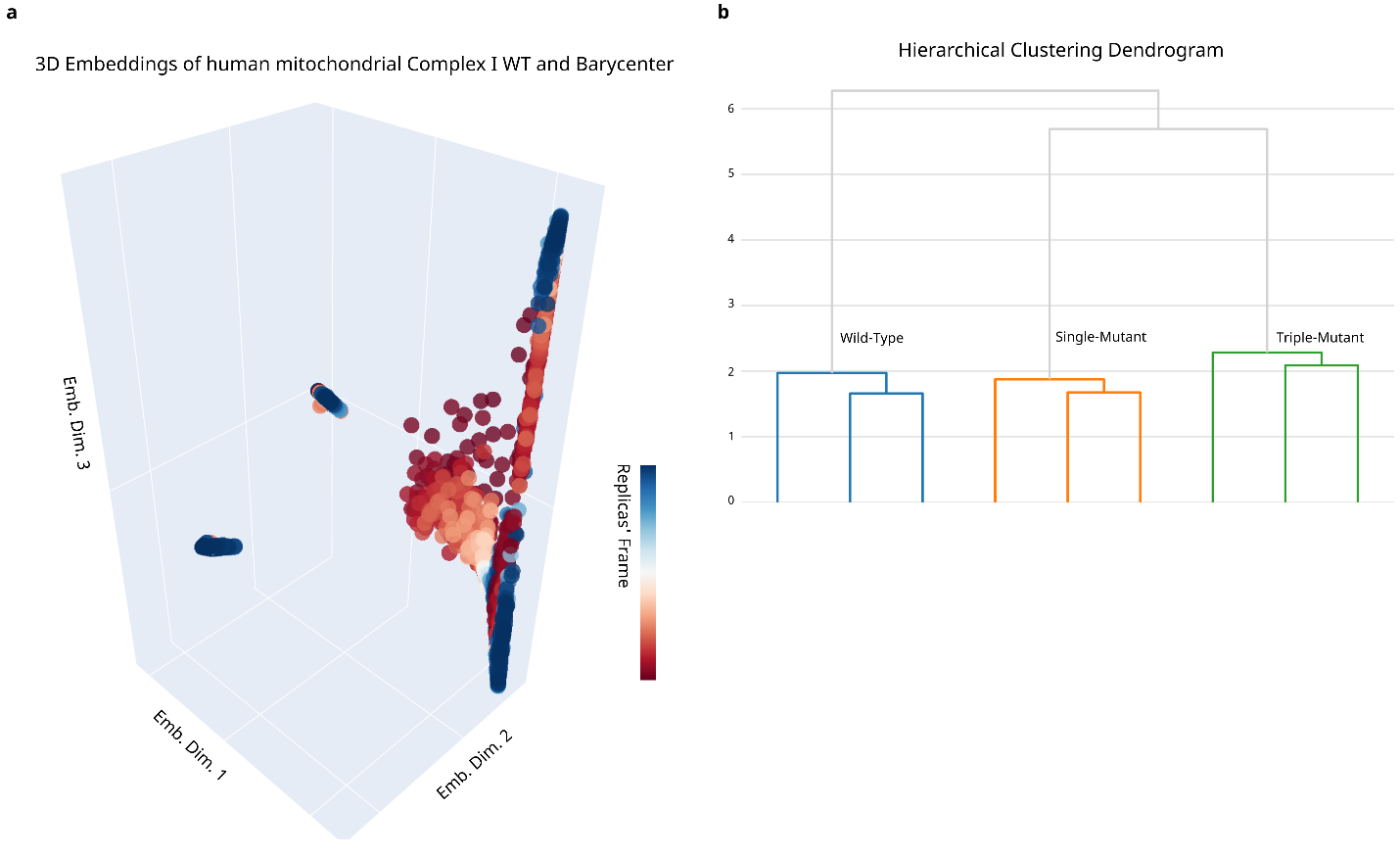
**

**Figure S2.** (a) Combined 3D embeddings of coarse-grained simulations of the wild‐type, single mutant, and triple mutant. (b) Final hierarchical clustering dendrogram computed on the combined embeddings, illustrating the three major branches corresponding to the wild-type and the mutants; the triple mutant system is the farthest from the wild-type.


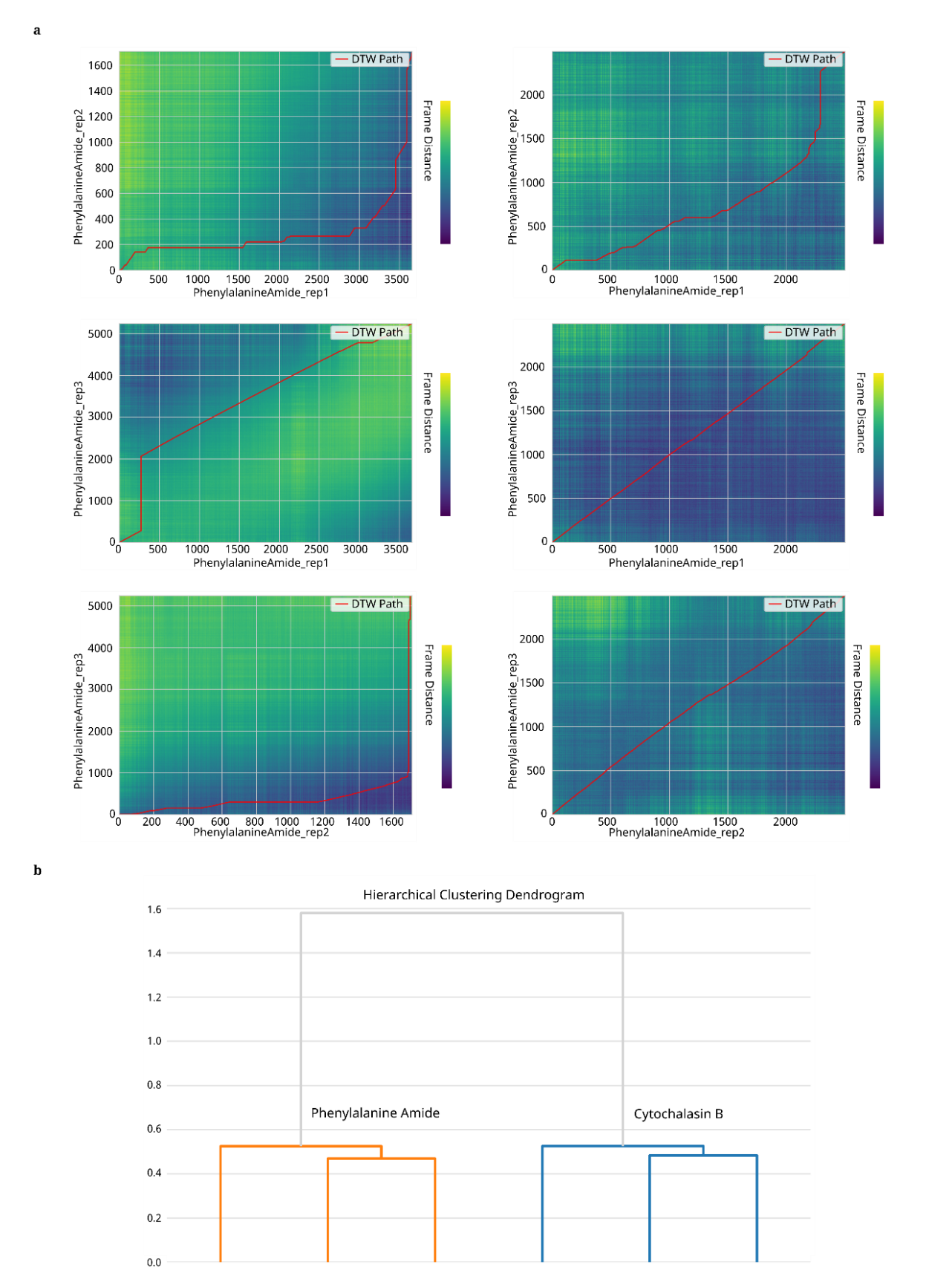


**Figure S3.** (a) Dynamic time–warping (DTW) alignments of ligand trajectories from SuMD (left column) and GaMD (right column) simulations. Each heatmap shows the pairwise frame distances between three independent replicates (rep1 vs. rep2, rep1 vs. rep3, and rep2 vs. rep3) of the phenylalanine amide system, with the optimal DTW path overlaid in red. In SuMD, unequal trajectory lengths produce pronounced deviations of the red path from the diagonal (time warping), whereas in GaMD (in which all trajectories have the same length), the DTW path remains nearly linear. (b) Hierarchical clustering dendrogram: All SuMD+GaMD replicates from the phenylalanine amide system (orange) form one cluster, whereas those from cytochalasin B (blue) form the other, illustrating that the learned embedding robustly discriminates between the two distinct inhibitor–protein systems.

**
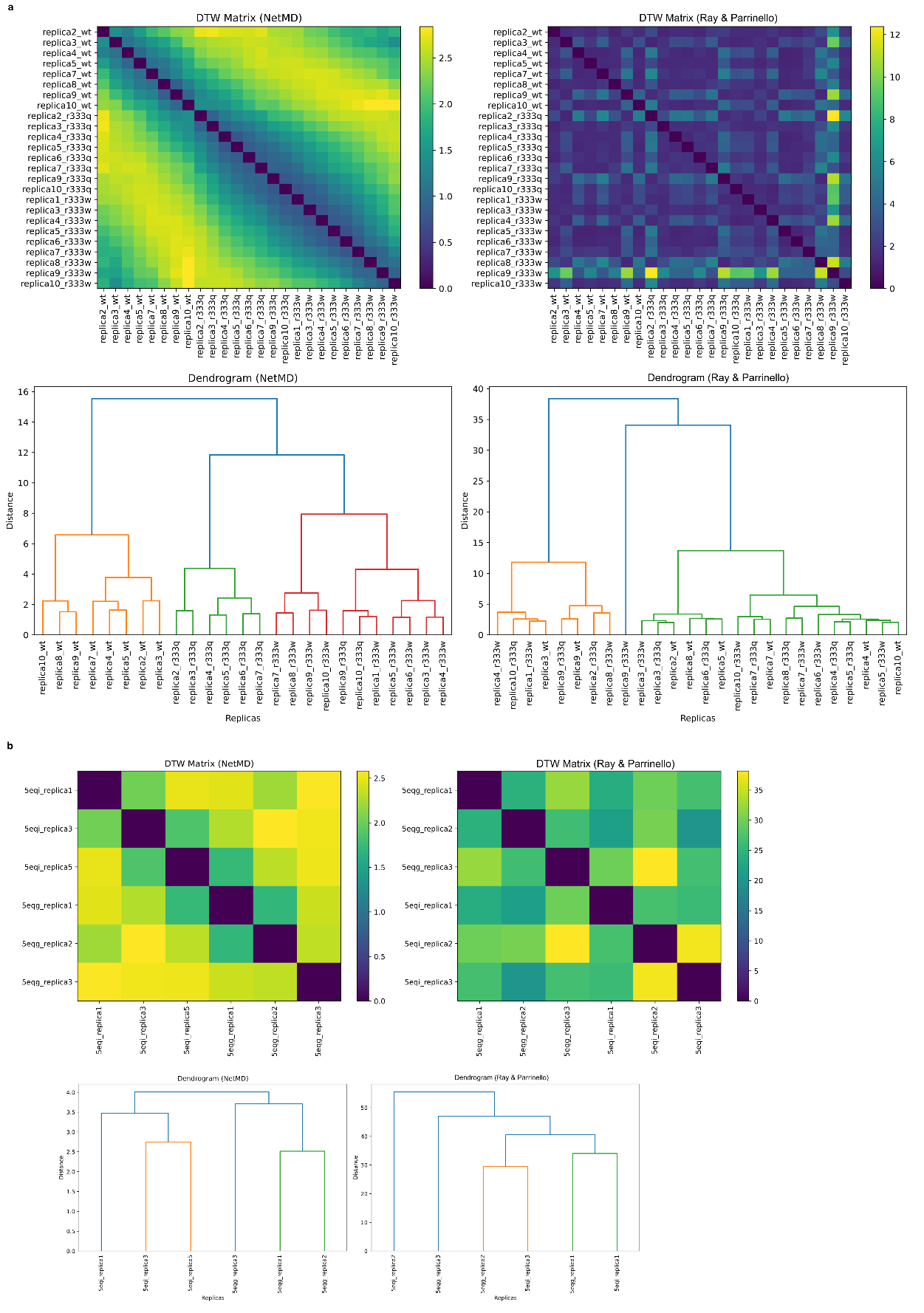
**

**Figure S4.** Distance heatmaps and dendrograms calculated on NetMD (left) and Ray&Parrinello (right) alignments for (a) GLUT1 wild-type and mutant systems and (b) GLUT1 ligand-binding systems.

# **Supplementary Videos**

## **Supplementary Video 1. Typical three-stage glucose transport cycle of GLUT1 protein**

The three-stage glucose transport cycle of GLUT1 comprises an initial intake phase in which glucose approaches and binds to the protein from the extracellular side, a central conformational transition driving the outward-occluded to inward-open switch, and a final release phase where glucose exits toward the cytoplasm. The video refers to the barycenter trajectory obtained from the *wild-type* molecular dynamics simulations.

# **Software Availability**

**Data availability**

The residue–residue contact files extracted from the MD trajectories of the GLUT1, KDM6A, and GLUT1-inhibitors systems that were analyzed in this study are available at (21). The landing page of the GitHub repository associated with this paper is [<https://github.com/mazzalab/NetMD>]. The documentation is available at [<https://mazzalab.github.io/NetMD>].

**Code availability**

Code to reproduce the analyses performed in this study is provided as a Jupyter Notebook at [[https://github.com/mazzalab/NetMD](https://github.com/mazzalab/NetMD?tab=readme-ov-file#-interactive-analysis-notebook)] (**tutorial** folder).

# **SI References**

1. [D. Sabbadin, S. Moro, Supervised molecular dynamics (SuMD) as a helpful tool to depict GPCR-ligand recognition pathway in a nanosecond time scale. *J Chem Inf Model* **54**, 372–376 (2014).](http://paperpile.com/b/dMCur1/xTZG)

2. [J. Schlitter, M. Engels, P. Krüger, Targeted molecular dynamics: a new approach for searching pathways of conformational transitions. *J Mol Graph* **12**, 84–89 (1994).](http://paperpile.com/b/dMCur1/CHSw6)

3. [S. Jo, T. Kim, V. G. Iyer, W. Im, CHARMM-GUI: a web-based graphical user interface for CHARMM. *J Comput Chem* **29**, 1859–1865 (2008).](http://paperpile.com/b/dMCur1/IpzRr)

4. [E. C. Meng, *et al.*, UCSF ChimeraX: Tools for structure building and analysis. *Protein Sci* **32**, e4792 (2023).](http://paperpile.com/b/dMCur1/xS4rK)

5. [D. A. Case, *et al.*, The Amber biomolecular simulation programs. *J Comput Chem* **26**, 1668–1688 (2005).](http://paperpile.com/b/dMCur1/h22VX)

6. [Y. Miao, V. A. Feher, J. A. McCammon, Gaussian Accelerated Molecular Dynamics: Unconstrained Enhanced Sampling and Free Energy Calculation. *J Chem Theory Comput* **11**, 3584–3595 (2015).](http://paperpile.com/b/dMCur1/shQ3F)

7. [F. Petrizzelli, *et al.*, Mechanisms of pathogenesis of missense mutations on the KDM6A-H3 interaction in type 2 Kabuki Syndrome. *Comput Struct Biotechnol J* **18**, 2033–2042 (2020).](http://paperpile.com/b/dMCur1/9MZCW)

8. [A. Liwo, *et al.*, Theory and Practice of Coarse-Grained Molecular Dynamics of Biologically Important Systems. *Biomolecules* **11** (2021).](http://paperpile.com/b/dMCur1/CXuUV)

9. [S. Menniti, E. Castagna, T. Mazza, Estimating the global density of graphs by a sparseness index. *Appl. Math. Comput.* **224**, 346–357 (2013).](http://paperpile.com/b/dMCur1/1O1xL)

10. [L. Rigobello, *et al.*, A computational study to assess the pathogenicity of single or combinations of missense variants on respiratory Complex I. (2023).](http://paperpile.com/b/dMCur1/s62HE)

11. [Y. Li, J. Sun, D. Li, J. Lin, The full activation mechanism of the adenosine A receptor revealed by GaMD and Su-GaMD simulations. *Proc Natl Acad Sci U S A* **119**, e2203702119 (2022).](http://paperpile.com/b/dMCur1/B4yu8)

12. [M. Karplus, J. Kuriyan, Molecular dynamics and protein function. *Proc Natl Acad Sci U S A* **102**, 6679–6685 (2005).](http://paperpile.com/b/dMCur1/8M0h2)

13. [A. Glielmo, *et al.*, Unsupervised Learning Methods for Molecular Simulation Data. *Chem Rev* **121**, 9722–9758 (2021).](http://paperpile.com/b/dMCur1/aovXV)

14. [A. Narayanan, *et al.*, Graph2vec: Learning distributed representations of graphs. *arXiv [cs.AI]* (2017).](http://paperpile.com/b/dMCur1/J8Ivy)

15. [B. Rozemberczki, O. Kiss, R. Sarkar, Karate Club: An API oriented open-source Python framework for unsupervised learning on graphs. *arXiv [cs.LG]* (2020).](http://paperpile.com/b/dMCur1/95qCT)

16. [Q. V. Le, T. Mikolov, Distributed representations of sentences and documents. *arXiv [cs.CL]* (2014).](http://paperpile.com/b/dMCur1/Cn6gi) Available at <https://arxiv.org/abs/1405.4053>.

17. [T. Mikolov, K. Chen, G. Corrado, J. Dean, Efficient estimation of word representations in vector space. *arXiv [cs.CL]* (2013).](http://paperpile.com/b/dMCur1/kpf6x) Available at <https://arxiv.org/abs/1301.3781>.

18. [F. Petitjean, A. Ketterlin, P. Gançarski, A global averaging method for dynamic time warping, with applications to clustering. *Pattern Recognit.* **44**, 678–693 (2011).](http://paperpile.com/b/dMCur1/jnRQQ)

19. [D. Schultz, B. Jain, Nonsmooth analysis and subgradient methods for averaging in dynamic time warping spaces. *Pattern Recognit.* **74**, 340–358 (2018).](http://paperpile.com/b/dMCur1/VCDZM)

20. [D. Müllner, Modern hierarchical, agglomerative clustering algorithms. *arXiv [stat.ML]* (2011).](http://paperpile.com/b/dMCur1/Kpvpw) Available at <https://arxiv.org/abs/1109.2378>.

21. [M. Mangoni, *et al.*, Synchronizing graph-embedded molecular dynamics trajectories via time-warping. (2025).](http://paperpile.com/b/SAn4n9/aZ5nS) Available at <https://doi.org/10.5281/zenodo.15970688>.
